# Asymmetric expression patterns reveal a strong maternal effect and dosage compensation in polyploid hybrid fish

**DOI:** 10.1186/s12864-018-4883-7

**Published:** 2018-07-03

**Authors:** Wuhui Li, Junmei Liu, Hui Tan, Lingling Luo, Jialin Cui, Jie Hu, Shi Wang, Qingfeng Liu, Fangzhou Hu, Chenchen Tang, Li Ren, Conghui Yang, Rurong Zhao, Min Tao, Chun Zhang, Qinbo Qin, Shaojun Liu

**Affiliations:** 1 State Key Laboratory of Developmental Biology of Freshwater Fish, Hunan Normal university, Changsha, 410081 Hunan People’s Republic of China; 2 College of Life Sciences, Hunan Normal University, Changsha, 410081 Hunan People’s Republic of China

**Keywords:** Dosage compensation, Duplicated genes, Hybridization, Maternal effect, Transcriptome

## Abstract

**Background:**

Hybridization and polyploidization are regarded as the major driving forces in plant speciation, diversification, and ecological adaptation. Our knowledge regarding the mechanisms of duplicated-gene regulation following genomic merging or doubling is primarily derived from plants and is sparse for vertebrates.

**Results:**

We successfully obtained an F1 generation (including allodiploid hybrids and triploid hybrids) from female *Megalobrama amblycephala* Yih (BSB, 2n = 48) × male *Xenocypri davidi* Bleeker (YB, 2n = 48). The duplicated-gene expression patterns of the two types of hybrids were explored using RNA-Seq data. In total, 5.44 × 10^8^ (69.32 GB) clean reads and 499,631 assembled unigenes were obtained from the testis transcriptomes. The sequence similarity analysis of 4265 orthologs revealed that the merged genomes were dominantly expressed in different ploidy hybrids. The differentially expressed genes in the two types of hybrids were asymmetric compared with those in both parents. Furthermore, the genome-wide expression level dominance (ELD) was biased toward the maternal BSB genome in both the allodiploid and triploid hybrids. In addition, the dosage-compensation mechanisms that reduced the triploid expression levels to the diploid state were determined in the triploid hybrids.

**Conclusions:**

Our results indicate that divergent genomes undergo strong interactions and domination in allopolyploid offspring. Genomic merger has a greater effect on the gene-expression patterns than genomic doubling. The various expression mechanisms (including maternal effect and dosage compensation) in different ploidy hybrids suggest that the initial genomic merger and doubling play important roles in polyploidy adaptation and evolution.

**Electronic supplementary material:**

The online version of this article (10.1186/s12864-018-4883-7) contains supplementary material, which is available to authorized users.

## Background

Hybridization and polyploidization are the driving forces of genomic evolution and speciation resulting from the instantaneous merger or doubling of two or more sets of divergent genomes [1, 2]. Generally, hybridization and polyploidy are two separate processes; however, these processes occur simultaneously to form an allopolyploid. A previous study reported that more than 25% of plants and 10% of animals have undergone hybridization and polyploidization in nature [3]. Allotriploidy and allodiploidy are rarely observed in lower vertebrates, expect for fishes, and triploid and diploid fish derived from hybridization, such as Oncorhynchus [4], Cyprinidae fish [5–7], and loaches [8], have been widely reported. Hybridization between populations or species can have pronounced fitness consequences. However, knowledge regarding how hybridization affects genomic reconciliation and gene regulation is limited.

The reunion of two divergent genomes in a common nucleus during allopolyploid speciation can lead to immediate and profound genome modifications, such as sequence elimination [9–13], epigenetic diversity [14–16], activation of genes and retroelements [17], and homoeologous interactions and exchanges [18, 19]. Notably, these modifications may directly or indirectly contribute to the establishment of a nascent polyploid and its evolutionary success as a new species [20]. In addition, the reconciliation of divergent parental genomes and regulatory interactions can be due to the initiation of hybrid heterosis [21]. Over generations, genetic and epigenetic changes are subject to selection and adaptation, and additional genes may be activated for allopolyploids to occupy an environmental niche [22]. Previous studies have documented that some important advantageous quality traits, such as vegetable and oil production [23], seed glucosinolate content [24], and cotton long fibers [25], resulted from subgenomes undergoing favorable selection during allopolyploid formation and domestication.

Transcriptome shock, which is another feature generating great interest in allopolyploids, refers to a sudden change in gene expression following the mixing of two dissimilar genomes with their own individual sets of transcription factors and chromatin profiles [26–28]. In particular, whether the two original parental genomes equally contribute to the gene expression levels either temporally or spatially during hybrid development remains unclear. To better understand the expression response of duplicate gene pairs, three patterns, including expression level dominance (the total level of expression for both homoeologs, ELD), homoeolog expression bias (relative contribution of homoeologs to the transcriptome, HEB), and homoeolog expression silence (where one or both paternal homoeologs have no expression) [29–31], have been recently described. Many studies have observed these expression patterns, including in cotton [29, 30], cyprinid fish [32, 33], *Tragopogon miscellu* [34], and *Arabidopsis lyrata* [35]. In addition, the influence of ploidy increases on gene regulation is still poorly studied in vertebrates, except for the gene-copy silencing attributed to complex dosage-compensation mechanisms observed in *Squalius alburnoides* [36], triploid Chinook salmon [37], and the triploid of *Ctenopharyngodon idellus* × *Megalobrama amblycephala* [38]. Although odd and even dosage effects could vary in a ploidy series, the coexpression and coevolution of orthologs and paralogs suggest that a selective advantage is obtained from the dosage dependency [39, 40]. Our understanding of the global expression patterns in allopolyploids is increasing; however, these expression patterns have not been well studied in different ploidy hybrids, especially F_1_ hybrids including an allodiploid and allotriploid hybrid.

In our previous study, two types of hybrids in the F1 generation, i.e., an allodiploid (2nBY, 2n = 48) and a triploid (3nBY, 3n = 72) hybrid, were successfully obtained from inter-subfamily hybridization between female blunt snout bream (*Megalobrama amblycephala* Yih, BSB, 2n = 48) and male *Xenocypris davidi* (*Xenocypri davidi* Bleeker, YB, 2n = 48) [5]. These two types of hybrids showed faster growth rates (> 10%) than both parents. In addition, the two types of hybrids exhibited phenotypic divergence, especially in regard to body height, which was statistically greater than that of the paternal YB (*p* < 0.05, Fisher’s exact test) [5]. Therefore, these two types of hybrids are ideal models for investigations of the relationship between duplicate gene expression and biological characteristics. In the present study, we successfully obtained the testis transcriptomes from the two types of hybrids and both parents. We determined the extent and direction of the homoeolog expression bias and overall gene expression differences among the different ploidy hybrids. Furthermore, we evaluated the effects of ploidy increases on gene regulation and their impact on the evolutionary potential of populations.

## Results

### Transcriptome assembly and annotation

To examine the changes in the global transcriptomic profile, we obtained twelve testis transcriptomes from two parents, i.e., BSB and YB, and their F1 hybrids,i.e., 2nBY and 3nBY. A combined total of 5.44 × 10^8^ (69.32 GB) clean reads was generated following the initial adapter trimming and quality filtering. Then, the twelve paired-end clean reads were assembled using a de novo method. We obtained 340,963 unigenes, and the numbers of unigenes (≥1000 bp) were 19,496 in BSB, 14,028 in YB, 21,617 in 2nBY, and 14,806 in 3nBY. In addition, the transcripts from all twelve samples were clustered by CD-HIT, which yielded a total of 499,631(261.8 Mb) reference sequences, with an N50 of 634 bp. These data are summarized in Table 1.

**Table 1:** Basic information of the transcriptome from the four groups of fish

Sample	Raw Reads	Clean reads	Clean bases(GB)	Q20 (%)	GC (%)	N50	NO. of unigenes	Total unigene base
2nBY1	45189626	44269784	6.64	96.72	47			
2nBY2	37142390	36318626	5.45	97	46.94			
2nBY3	51166402	50931544	4.69	97.03	47.14	1144	102298	74506397
YB1	41884596	40109404	6.02	96.45	46.66			
YB2	43785474	41894952	6.28	96.84	47.03			
YB3	54137964	54016980	4.94	97.88	45.13	886	79482	49222867
3nBY1	41129920	40255526	6.04	96.64	46.68			
3nBY2	47209846	46103658	6.92	95.63	46.78			
3nBY3	50808026	50715054	4.63	98.03	46.67	968	81212	52695486
BSB1	47315502	45193216	6.78	96.57	47.04			
BSB6	43210552	41392052	6.21	96.43	46.82			
BSB7	52496020	52413280	4.72	97.81	46.78	1286	77971	62344165
Total	555476318	543614076	69.32	96.92	46.722	634	499631	261825267

Of the 499,631 assembled contigs, 225,203 (45.07%) contigs had significant alignments (e-value<1e-5) to known proteins in the public databases UniProt (Swiss-Prot and PFAM) and NCBI (Nr). In total, 364,543(72.96%) unigenes were annotated in at least one database (KO, KEGG, Nt, and KOG), while 135,088 (27.04%) sequences showed no or poor similarity matches and may represent specific transcripts with unknown functions (Additional file 1: Table S1).

### Merged genomes are dominantly expressed in different ploidy hybrids

**Fig. 1:**
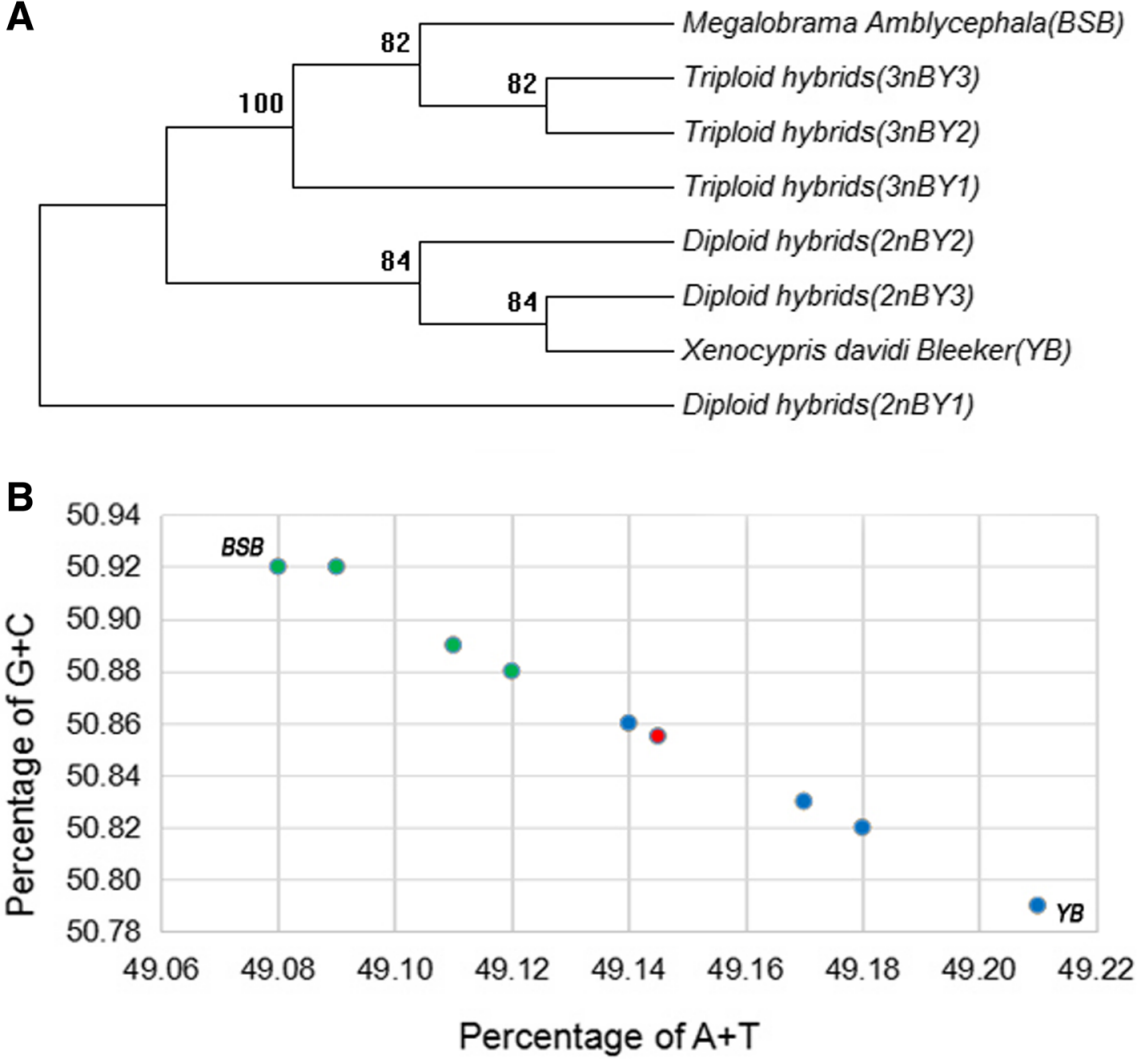
Interaction and expression of homoeologs in eight orthologs. **a** A maximum likelihood tree constructed from 4265 orthologs showing the sequence similarity between the two types hybrids and the original parents. **b** Percentages of A + T and C + G bases in the eight linked orthologous sequences. Blue dot  represents three biological repeats of allodiploid hybrids, and green dot represents three biological repeats of triploid hybrids

As the parent genomes coexist in all somatic cells of the 2nBY and 3nBY hybrids, a DNA sequence analysis can clearly distinguish the orthologous origin of a gene from either parent based on special high-density single nucleotide polymorphisms (SNPs). In total, 22.4 Mb of orthologous sequences, including 4265 genes (CDS or partial CDS), were obtained after reciprocal BLAST and removal of the redundant and unmatched paired-end bases. Then, the orthologous sequences were linked into eight long sequences, an maximum likelihood tree was constructed to evaluate sequence similarity. The tree showed that the 4265 orthologs from 2nBY shared a high sequence identity with the YB genome, while 3nBY shared a high sequence identity with the BSB genome (from 97.6 to 99.5%) (Fig. 1a, Table 2). Our results indicated that merged parent genomes were dominantly expressed in different ploidy hybrids. The orthologous data are displayed in Additional file 2.

**Table 2:** Sequence similarity among the eight linked long orthologs

	BSB	3nBY1	3nBY2	3nBY3	2nBY1	2nBY2	2nBY3	YB
BSB	1.000							
3nBY1	0.995	1.000						
3nBY2	0.993	0.992	1.000					
3nBY3	0.994	0.993	0.993	1.000				
2nBY1	0.987	0.989	0.986	0.986	1.000			
2nBY2	0.985	0.986	0.986	0.987	0.987	1.000		
2nBY3	0.985	0.986	0.986	0.986	0.987	0.991	1.000	
YB	0.976	0.979	0.977	0.977	0.987	0.985	0.986	1.000

In addition, the percentages of the bases A, T, C, and G bases were determined in the eight linked long sequences (Additional file 1: Table S2). No significant difference was observed among the eight linked long sequences; however, the percentages of (A + T) and (C + G) clearly showed that the 3nBY orthologs were closer to the BSB genome, while 2nBY orthologs were closer to the YB genome (Fig. 1b).

### Asymmetric expression patterns in the two types of hybrids

**Fig. 2:**
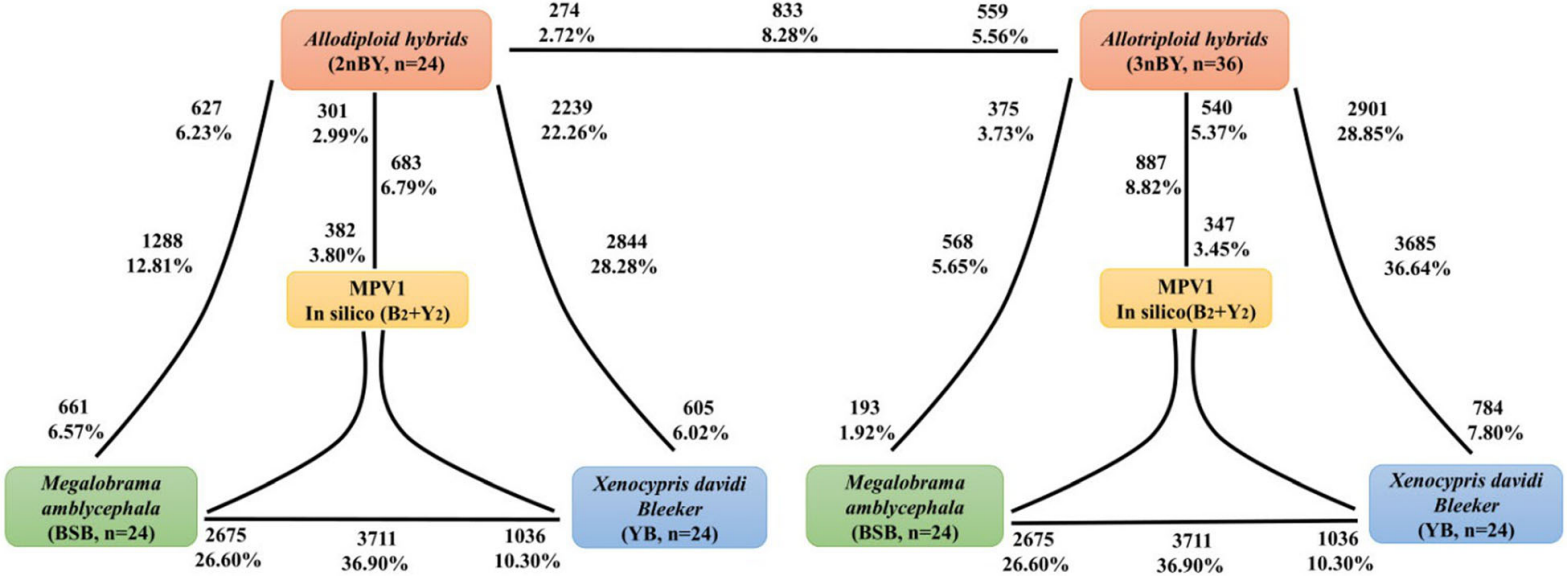
Differentially expressed genes between each of the two types of hybrids and their origin parents. Bold text indicates the total number and fraction of genes that are differentially expressed in each comparison. The total number of differentially expressed genes that were upregulated in each comparison is also shown. For example, 3711 (36.90%) genes are differentially expressed between BSB and YB. Of these genes, 2675 (26.60%) genes are upregulated in BSB, and 1036 (10.30%) genes are upregulated in YB

To detect the differentially expressed genes (DEGs), a false discovery rate (FDR) < 0.001 and an absolute value of the log 2-fold change > 1.0 were used as thresholds in the comparisons between the parents and the different ploidy-level hybrids. In total, 10,057 unigenes (including 7890 annotated genes) were found to be coexpressed in the parents and the two types of hybrids (Additional file 3). The comparison of the expression levels in the two parents revealed that 2675 genes were upregulated in the maternal BSB, and 1036 genes were upregulated in the paternal YB (Fig. 2). Then, the two hybrids were compared with both parents, reveling that the numbers of DEGs were asymmetric (*p* < 0.05; Fisher’s exact tests) (Fig. 2). For example, 2239 (22.26%) genes were upregulated and 605 (6.02%) genes were downregulated in 2nBY compared with those in the paternal YB, while 627 (6.23%) genes were upregulated and 661 (6.57%) genes were downregulated compared with those in the maternal BSB. In 3nBY, 357 (3.73%) genes exhibited increased expression levels and 193 (1.92%) genes exhibited decreased expression levels compared with those in the maternal BSB, while 2901 (28.85%) genes showed higher expression levels and 784 (7.80%) genes showed lower expression levels when compared those in parental YB. The numbers of DEGs in the two types of hybrids that exhibited asymmetric expression when compared with those in the parents, indicate that genomic merger and doubling have a large effect on the expression of duplicate genes, and the effect of the maternal BSB genome is larger than the effect of the paternal YB genome.

### Maternal effect and expression level dominance

**Fig. 3:**
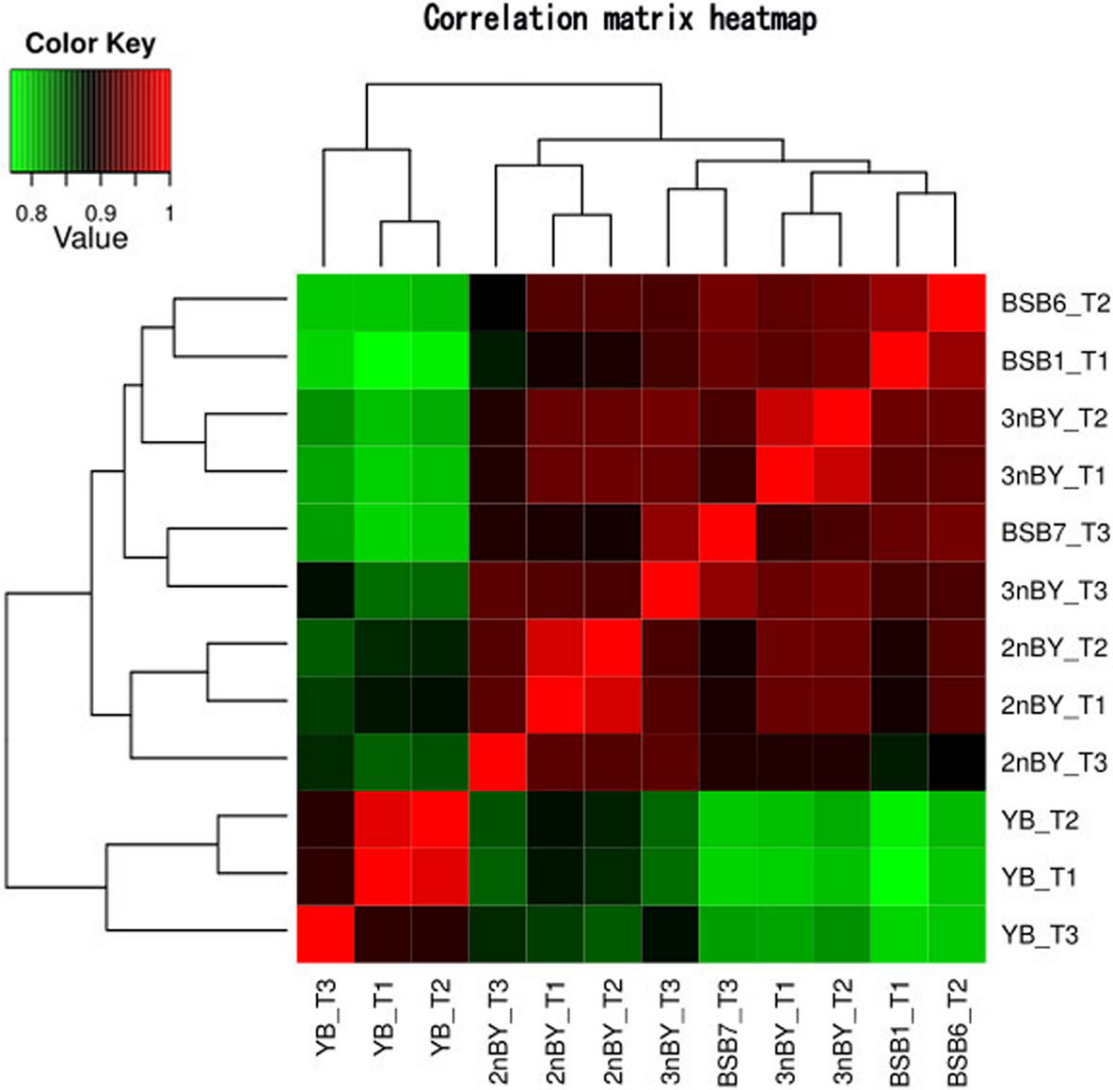
Global expression levels of all coexpressed genes between hybrids and parents. A correlation matrix heatmap of 10,057 coexpressed unigenes between the two types of hybrids and the original parents

**Fig. 4:**
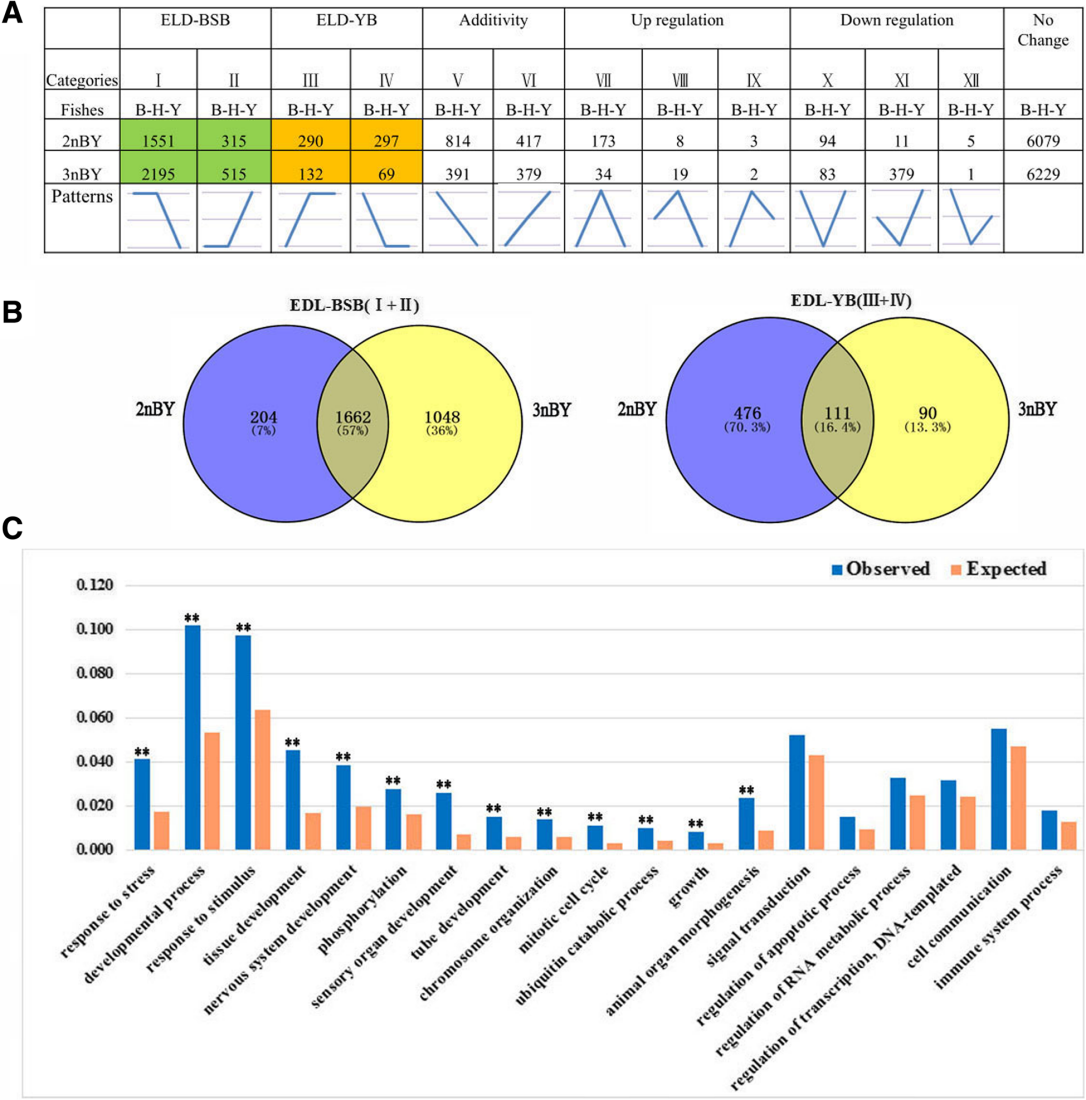
Partitioning of expression patterns in the two types of hybrids. **a** The 12 possible differential expression states in the allodiploid and triploid hybrids relative to their diploid parents. The green box shows the BSB-ELD genes, and the orange box shows the YB-ELD genes. **b** Dominant expression gene inheritance between 2nBY and 3nBY. **c** GO functional classification of ELD genes in 2nBY and 3nBY. ** *p* < 0.05

To better understand the effect of genomic merger and doubling on global expression patterns, the expression level dominance (ELD) of the duplicated genes in the two types of hybrids was investigated. First, we generated a correlation matrix of all coexpressed genes at the global expression level (Fig. 3). The expression levels of the overall genes indicated that both 2nBY and 3nBY were more closely related to the maternal BSB than to the paternal YB. Then, we divided these genes into 12 categories, based on their differential expression patterns by comparing the hybrids to their parents according to a method previously described by Rapp et al. (2009) (Fig. 4a). We classified the ELD genes in 2nBY and 3nBY that showed expression levels similar to those in BSB as BSB-ELD genes (categories I and II), and ELD genes with expression levels equivalent to those observed in YB were labeled YB-ELD genes (categories III and IV). Among the 10,057 shared unigenes, 2453 and 2911 genes demonstrated parent-ELD in 2nBY and 3nBY, respectively (Fig. 4a). In 3nBY, we determined that approximately 2710 (26.95%) genes showed BSB-ELD, and 201 (2.09%) genes showed YB-ELD. In 2nBY, 1866 (18.55%) genes showed BSB-ELD, which exhibited more influence than paternal YB-ELD (587 genes, 5.84%). These results showed that the imbalance in the number of ELD genes compared with that in the original parents is inclined toward the maternal BSB genome in both types of hybrids. Furthermore, both the upregulated genes (categories VII, VIII, and IX) and the downregulated genes (categories X, XI, and XII) showed significant differences between the two types of hybrids (*p* < 0.05 in comparisons; Fisher’s exact test), including 184 upregulated genes and 110 downregulated genes in 2nBY and 55 upregulated genes and 463 downregulated genes in 3nBY.

We compared the ELD (I, II, III, and IV) and the differentially expressed genes (VII, VIII, IX, X, XI, and XII) in the two types of hybrids and found that most parental-ELD genes were heritable between 2nBY and 3nBY (Fig. 4b). Then, we performed a GO analysis (level 4) to determine the possible functions of the parental-ELD genes in 2nBY and 3nBY (Additional file 4). The parental-ELD genes were classified as active in the response to stress and stimuli, developmental processes, phosphorylation, tube development, growth, signal transduction, and cell communication (Fig. 4c). The genes related to growth, such as *gpc4*, *stat3*, *mgat5*, *ryk*, *vdac2*, and *fzd2*, demonstrated maternal BSB-ELD and exhibited greater expression levels than the paternal YB genes in the two types of hybrids (Additional file 5).

### Dosage compensation in triploid hybrids

**Fig. 5:**
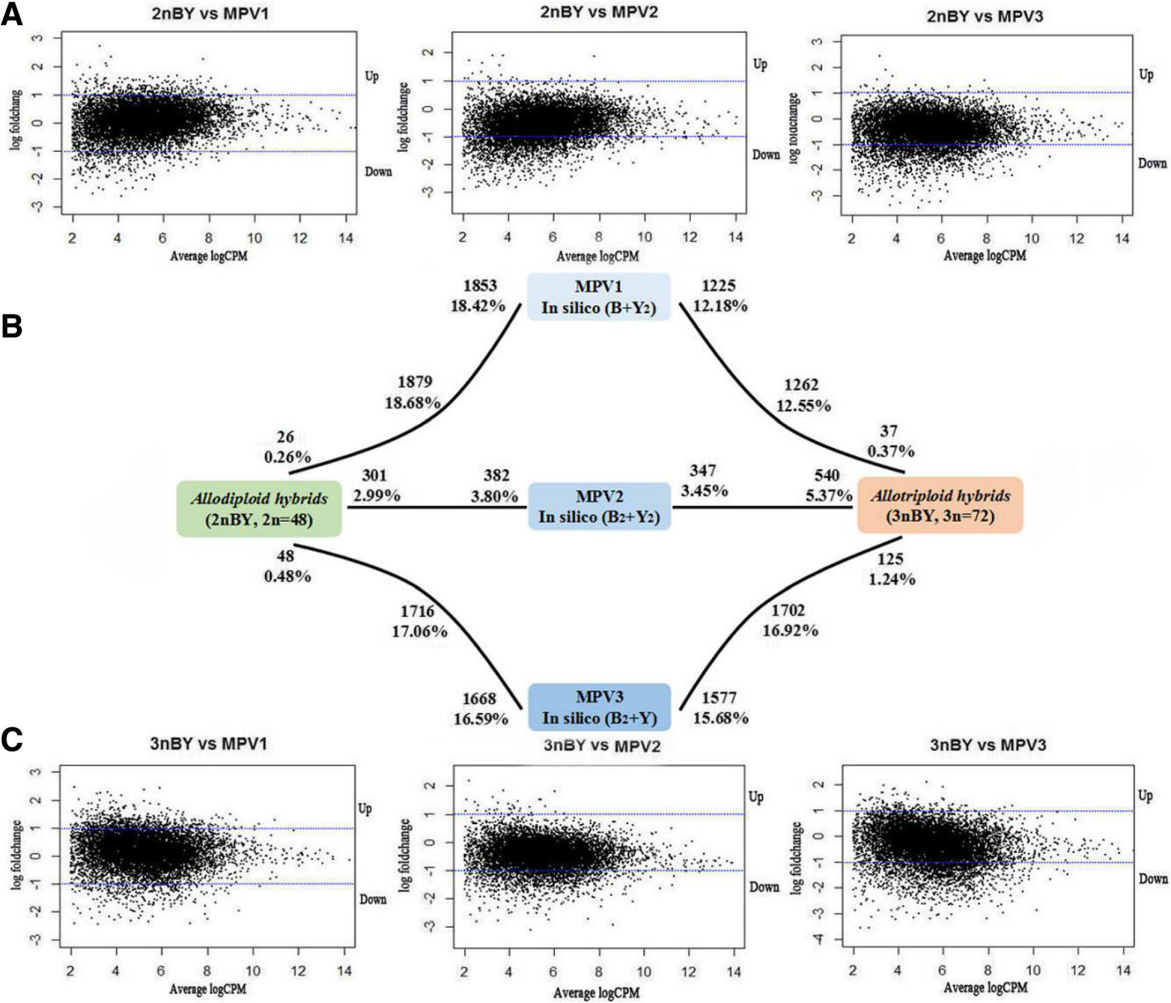
Distribution of differentially expressed genes between the two types of hybrids and MPVs. **a** and **c** The global expression of genes in 2nBY and 3nBY compared with MPVs. The black dots between the two blue lines represent genes with no significant difference in expression levels, and the other dots represent genes with significant differential expression levels (> 2-fold change and FDR < 0.05). **b** Bold text represents the total number and fraction of genes that are differentially expressed between the two types of hybrids, with predicted expression levels of mid-parents in the silico module of B + Y_2_, B_2_+ Y_2_, and B_2_ + Y

To understand the effects of ploidy increases on gene regulation and their impact on the evolutionary potential of populations, we compared the gene expression levels in 2nBY and 3nBY with Mid-parent values (MPVs, synthetic diploid and triploid), which were calculated based on the expression levels of the two parents. In 2nBY, 301 (2.99%) genes were upregulated and 382 (3.80%) genes were downregulated compared with those in the synthetic diploid hybrids (MPV2), while 26 (0.26%) and 48 (0.48%) genes were upregulated compared with those in the synthetic triploid hybrids MPV1 and MPV3, respectively, and 1853 (18.42%) and 1668 (16.59%) genes were downregulated compared with those in MPV1 and MPV3, respectively. Significantly more genes were downregulated than upregulated in the comparison of the 2nBY expression levels and those of the synthetic triploid hybrids (*p* < 0.05; Fisher’s exact tests) (Fig. 5). The number of DEGs between 2nBY and the synthetic polyploids indicates that genomic doubling may have a large influence on duplicate gene expression. In 3nBY, 540 (5.37%) genes were upregulated and 347 (3.45%) genes were downregulated compared with those in the synthetic diploid (MPV2). Additionally, 37 (0.37%) and 125 (1.24%) genes showed increased expression levels compared with those in the synthetic triploids MPV1 and MPV3, and 1225 (12.18%) and 1577 (15.68%) genes showed decreased expression levels in 3nBY compared with those in MPV1 and MPV3, respectively (Fig. 5). The global expression patterns of 2nBY and 3nBY were both biased towards MPV2, suggesting that 3nBY reduced its expression levels to levels similar to those of the diploid state.

### Sanger sequencing and real-time quantitative PCR (qPCR) validation

As the divergent parent genomes coexist in each nucleus, Sanger sequencing was performed to detected the genome interaction at the DNA level. The duplicate genes experienced strong interactions, such as *tcs22* and *myo18b*, which formed chimeras, and *fitm and ptpn,* which experienced an In-Del in the noncoding region. The results are shown in Additional file 6.

**Fig. 6:**
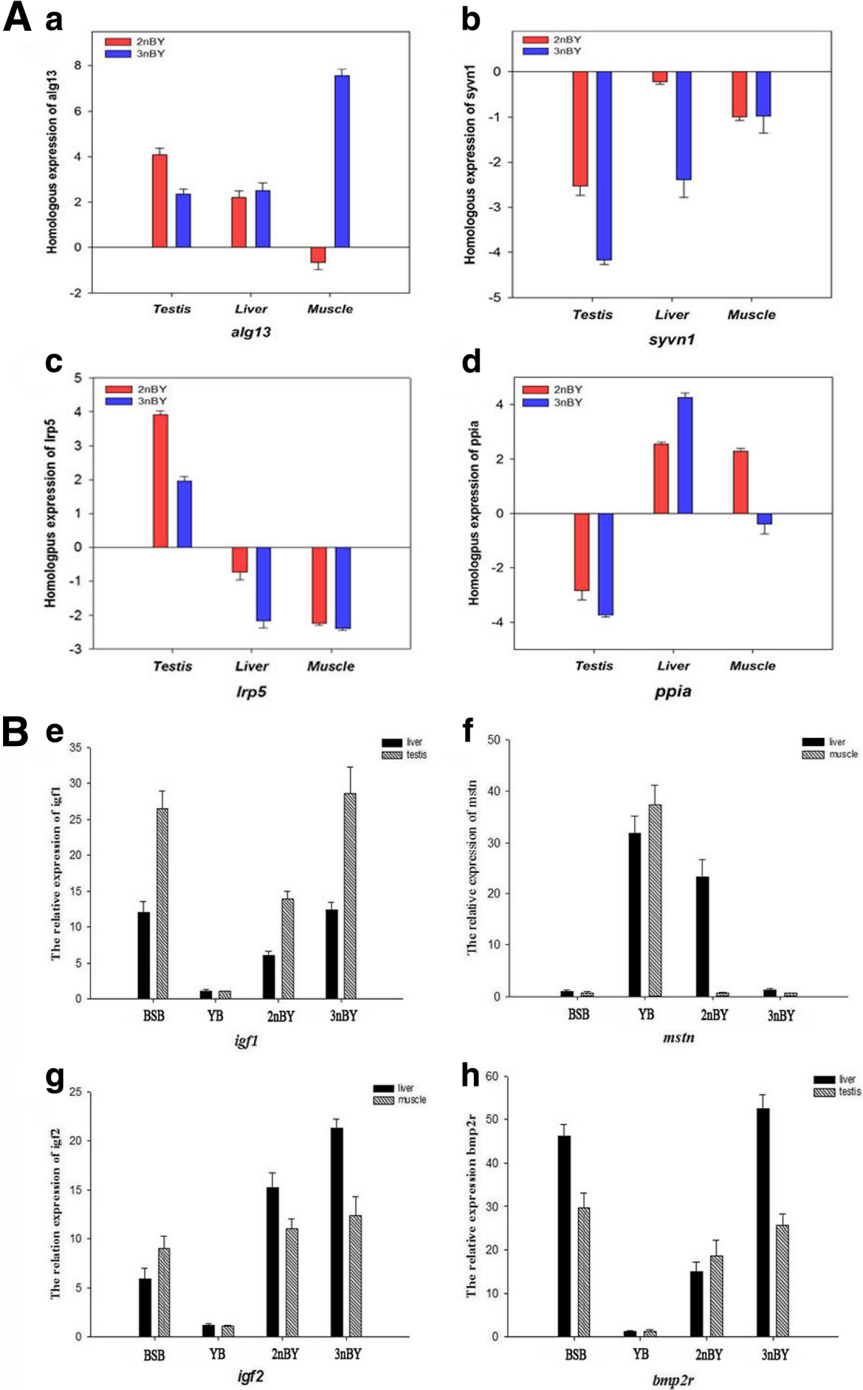
Quantitative real-time PCR analysis of HEB and ELD in different tissues. **A** HEB genes verified in different tissues (*alg13*, *syvn1*, *lrp5*, and *ppia*). The ΔCт value represents the relative expression levels of the BSB and YB homoeologs. A positive number represents YB-dominant genome expression, and a negative number represents BSB-dominant genome expression. **B** ELD genes verified in different tissues (*igf1*, *mstn*, *igf2*, and *bmp2r*) between the allodiploid and triploid hybrids

Real-time quantitative PCR (qPCR) is considered a suitable and effective method for detecting gene expression levels in different tissues; therefore, we performed qPCR explore the direction and extent of HEB genes (*syvn1*, *lrp5*, *ppia*, and *alg13*) in three tissues (liver, muscle and testis) using homoeolog-specific primers (Additional file 7: Figure S1). Interestingly, two scenarios were observed in the two types of hybrids: (1) the homoeolog gene expression uniformly showed bias toward one parent in all tested tissue or (2) the homoeolog genes in different tissues had different parental biases. For example, the expression of *syvn1* and *lrp5* was biased toward one parent in all three tissues in both 2nBY and 3nBY; *ppia*, *alg13*, and *lrp5* showed different paternal bias expression levels in different tissues in both 2nBY and 3nBY. In addition, *alg13* and *ppia*, in muscle, showed different paternal bias expression levels in 2nBY and 3nBY (Fig. 6a). The results indicate that the direction and extent of HEB were selective among different tissues in different ploidy hybrids. In addition, we also performed qPCR to detect the ELD in different tissues. The genes *igf1*, *igf2* and *bmp2r* in BSB and the two types of hybrids were expressed at greater levels than those in the paternal YB, while the *mstn* gene showed the opposite results (Fig. 6b).

## Discussion

### Divergent genome merger and asymmetric expression patterns

Recently, comparative genomics has indicated that the genome in progenies suffers a dynamic variation during hybridization and polyploidization, resulting in genomic incompatibility and transcriptome shock. The direct effects of genomic merger include genomic structural alterations, such as reciprocal homoeologous recombination, non-reciprocal transfer, and gene conversion [41, 42]. Generally, recombination has an impact on the genome evolution by affecting the efficacy of selection and promoting adaptation because selection acting across multiple loci becomes more efficient as the linkage between loci decreases [43]. Chimera formation, which is the most common genomic alternation, can occur rapidly in somatic cells during allopolyploid development and has been detected in many allopolyploids [17, 44]. In our study, the expressed duplicated genes in 2nBY shared a high sequence identity with those in YB, while 3nBY shared a high sequence identity with BSB; however, differences were observed, namely, some genes underwent alterations (Fig. 1, Additional file 1: Table S2). These alterations primarily included loci mutations, chimera formation (recombination), and loci duplication or replication. Sanger sequencing confirmed the presence of genomic alterations in the chimeras (*tcs22* and *myo18b*) and In-Dels in a noncoding region (*fitm* and *ptpn*) (Additional file 6). Sequence evolution and epigenetic regulation play interactive and pervasive roles in mediating the regulatory incompatibilities between divergent genomes, leading to natural variation and selective adaptation during allopolyploid evolution [15].

Recently, several studies have documented that genomic merger alters the transcriptome more than genome doubling in allopolyploid plants [45–47]. Another study revealed that genomic merger mainly affects HEB, and polyploidization mainly affects the global gene expression in a allotetraploid fish [32]. To better understand the impact of divergent-genome merger on the genome-wide alterations in gene expression at different polyploidy levels, we compared the expression levels of coexpressed genes between the two types of hybrids and their parents. The number of upregulated genes was nearly two times greater than the number of genes that were downregulated in BSB compared with YB, suggesting that the expression levels of BSB homoeologs were higher than those of YB homoeologs (Fig. 2). Comparing the gene expression levels to both parents, the number of DEGs in the different ploidy hybrids was asymmetric (Fig. 2). The numbers of DEGs between the hybrids and paternal YB were significantly more than those between the hybrids and maternal BSB. These results indicated that divergent-genome merger has a greater impact on global expression levels than genome doubling.

### Expression level dominance and homoeolog expression bias

Genomic merger and doubling could cause the immediate, massive, and situational disruptions in gene expression between parental copies of genes in different tissues [46, 48, 49]. The direction and extent of homoeolog expression bias and expression level dominance alterations are generally observed in hybridization through evolution at the polyploid level. In plants, genome-wide changes in gene expression levels are widely observed in synthetic and natural polyploids [29, 30, 45, 50, 51]. In teleosts, only a few intraspecific cross hybrids, including brook charr [52], rainbow trout [53], lake whitefish [54], and *Carassius auratus red var.* × *Cyprinus carpio* [32, 33], have been reported to display the genome dominance phenomenon. In the present study, the 12 categories of expression level patterns are described above (Fig. 4a). We detected a significantly greater number of ELD-BSB genes (1866 and 2710) than ELD-YB genes (587 and 199) in both 2nBY and 3nBY (*p* < 0.05, Fisher’s exact tests), which indicates that the maternal BSB homoeologs have a greater effect on the modification of expression level dominance categories than the paternal YB homoeologs. In addition, ELD genes were mostly shared between 2nBY and 3nBY, and the GO functions were similar in the two types of hybrids (Fig. 4b and c), which may result in the two types of hybrids sharing similar traits with the maternal BSB, such as appearance and growth level. Gene expression nonadditivity may play a crucial role in the adaptation and evolution of newly formed allopolyploid plants and animals [55]. In addition to parental ELD, another phenomenon was also described; middle-expression levels were found in the polyploids based on the relative expression levels of the two parents. In our study, 7310 (72.68%) genes in 2nBY and 6999 (65.59%) genes in 3nBY (in the additivity and no change categories) showed expression levels that were regulated by homoeologs from both parents (Fig. 4a). qPCR was performed to verify the expression levels of four growth-related genes, and *igf1*, *igf2*, and *bmp2r* showed maternal-BSB dominant expression, while the gene *mstn* showed paternal-YB dominant expression in different tissues (Fig. 6a). Expression level dominance may contribute to heterosis in polyploidy hybrids [38, 56, 57].

Duplicate gene pairs in polyploids may display homoeolog expression bias in which, the two homoeologs are expressed unequally and often at varying levels among different tissues compared with a priori expectations based on the progenitor diploid expression levels [29–33, 42]. In this study, the linked long sequences from 4265 orthologs revealed that the YB homoeologs were dominantly expressed in the testis of 2nBY, while the BSB homoeologs were dominantly expressed in the testis of 3nBY. These results indicate that the parent genomes underwent a strong dynamic selection during the development of different polyploidy levels. Additionally, qPCR was performed to detect the HEB genes (*syvn1*, *alg13*, *lrp5*, and *ppia*) in liver and muscle tissues and showed that the direction of HEB was selective among different tissues (Fig. 6a). Homoeolog expression bias may contribute to some advantageous traits of allopolyploids, such as growth regulation [32, 33, 38], improvement in biotic stress tolerance [34], and temperature adaption.

### Maternal effect and dosage compensation in different ploidy levels

Complex dosage-compensation mechanisms, such as the silencing of one of three sets of alleles, result in decreased transcript levels in the triploid to the diploid state, which has been observed in several triploid cyprinid fishes [38, 58, 59]. Additionally, maternal effects on morphological and life-history traits, such as seed mass and germination, have been well documented in plants [60]. However, the impact of genome doubling and the maternal effect on genome-wide alterations in gene expression are still rarely studied in fish with different ploidy levels due to the lack of ideal material. In the present study, the global expression levels of all coexpressed genes revealed that both 2nBY and 3nBY hybrids were biased towards the maternal BSB genome (Fig. 2). Comparing the expression levels of the coexpressed genes between the hybrids and MPVs (synthesis polyploidy), the number of DEGs did not significantly differ between the two types of hybrids and MPV2 (synthesis diploid), while the upregulated and downregulated genes were significantly different between the two types of hybrids and MPVs (synthesis triploid). Our results suggest that a strong maternal effect on gene expression exists in different ploidy hybrids, and the effect is reduced in triploids compared with that in diploids. However, we only determined the testis expression profile, and further experiments are required to determine whether the observed maternal effect persists over time and the degree to which this effect is manifested as differences in morphology or physiology. Dosage compensation could result in novel epigenetic regulation methods in triploid fishes [61] and contribute to heterosis in triploid maize [62].

## Conclusions

In this study, we explored the overall gene expression patterns in an allodiploid hybrid fish and a triploid hybrid fish using RNA-Seq reads. To better understand the effects of genomic merger and doubling on duplicate gene expression, we first used orthologs to explore homoeolog expression and showed a reverse direction of HEB in different ploidy hybrids. Then, the correlation matrix of global expression and analysis of 12 expression patterns (homoeolog dominance, up/downregulation, and mid-parents) revealed that both allodiploid and triploid hybrids showed maternal-BSB expression level dominance, indicating that a maternal effect strongly exists at different ploidy levels. In addition, we observed that the triploid hybrid had reduced expression levels compared to those of the diploid hybrid, indicating the existence of complex dosage-compensation mechanisms following genomic doubling. The determined expression patterns (HEB, ELD, maternal effect, and dosage) following divergent-genome merger and doubling obviously play important roles in polyploidy speciation and adaptation; however, other expression regulation mechanisms require further investigation.

## Methods

### Animal materials

The natural BSB were obtained from Liangzi Lake (Hubei Province), the natural YB were obtained from Dongting Lake (Hunan Province), and all cultured in ponds at the Engineering Center of Polyploidy Fish Breeding of the National Education Ministry located at Hunan Normal University, China. During the breeding season (May to June 2015), the first-generation hybrids were obtained from the hybridization of female BSB × male YB, and the protocols used for the crossing and culturing have been previously described [5]. All larvae were fed in the same pond at a suitable water temperature and dissolved oxygen content at the Engineering Center of Polyploidy Fish Breeding of the National Education Ministry located at Hunan Normal University, China. After two years, we harvested the fish for experimentation, including the parents BSB and YB and two types of hybrids (allodiploid and triploid hybrids). The ploidy types of the two hybrids were confirmed based on their DNA contents using a flow cytometer (cell counter analyzer: Partec) [63]. All experimental fishes (each group contained three biological repeats) were euthanized using 2-phenoxyethanol (Sigma) prior to dissection. The testis tissues were excised and immediately placed into RNALater (AM7021, Ambion Life Technologies, Carlsbad, CA, USA), according to the manufacturer’s instructions, frozen by liquid nitrogen, and stored in a − 80 °C freezer.

### RNA extraction, sequencing, assembly and annotation

The total RNA was extracted from the testicular tissues using TRIzol reagent (Invitrogen) according to the standard TRIzol protocol. Agarose (1.0%) gel electrophoresis and measurements of the optical density at 260 nm (OD260/OD280 ratio) were performed to assess the RNA quality. A TURBO DNA-free kit was used to remove DNA contamination. The first and second strands of cDNA were synthesized using a random hexamer primer, M-MuLV Reverse Transcriptase (RNase H-), and dNTPs. cDNA fragments 150~ 200 bp in length were collected and purified with an AMPure XP system (Beckman Coulter). Then, the short fragments were used to perform PCR amplification and construct libraries. Finally, the twelve libraries were sequenced on an Illumina Hiseq 2000/2500 platform.

After obtaining the raw read database, reads with only adaptations and low-level reads (unknown nucleotides > 5%) were removed using the FastQC software (Babraham Bioinformatics) [64]. Then, the de novo assembly was performed using Trinity, with the min_kmer_cov set to 2 by default and all other parameters set to the default levels [65]. The short reads were assembled into longer contigs based on their overlapping regions. Blastx alignment (e-value<1e-5) between the assembled unigenes and protein databases (NR, Swiss-Prot, KEGG and COG) was employed to annotate the contigs. The best alignment results were assigned as the annotations of the unigenes. In cases of conflicting results among the databases, the priority order was defined as Nr, Swiss-Prot, KEGG, and COG. In addition, the Blast2GO program was used to obtain the Gene Ontology (GO) annotations [66].

### Mapping and differential expression

To obtain the shared transcripts among the four species, reference transcripts were created by the merging the transcripts of BSB, YB, 2nBY and 3nBY contigs, using CD-HIT at a threshold of 95% [67]. We utilized the merged sequences as the reference transcripts because this database was built using transcripts from both the parents and the two types of hybrids. Then, the total clean reads were aligned against the merged sequences using Blat [68]. The transcript expression levels were calculated using the fragments per kilobase per million fragments mapped (FPKM) method. Finally, we used DEseq in R software, version 2.13 (R Foundation for Statistical Computing, Vienna, Austria) [69], to search for DETs using a false discovery rate (FDR) < 0.001 and a threshold normalized absolute log 2-fold change > 1.0. Differential expression was assessed in the two types of hybrids and their parents using Fisher’s exact tests [70].

### Analyses of expression level dominance

To better explore expression level dominance, we first removed those transcripts with 0 RPKM from the libraries of both the parents and the two types of hybrids. Then, genes that were identified as differentially expressed between the two types of hybrids and the parents were binned into the following 12 possible differential expression categories according to a previous study (Fig. 4a): parent ELD (I, II, III, and IV), mid-parents (V and VI), increased expression (VII, VIII, and IX) and decreased expression (X, XI, and XII). For each of the 12 categories listed above (which are based on the joint expression levels in both homoeologs), we calculated the RPKM values of the reads to examine the gene expression levels in each homoeolog pair. An FDR < 0.005 and an absolute value of log 2 ratio ≤ 1 were used as thresholds to determine the significance of the gene expression differences between two species.

### Subgenome domination and interaction

Following parent genome fusion and doubling in the two types of hybrids, the whole transcriptome sequences can reflect parent genome interaction at the mRNA levels. Here, reciprocal BLAST was used to determine the coexpressed genes (partial CDS) in six individuals and two parents at a cut-off e-value of <1e-6. A phylogenetic tree was generated to display the selectively expressed genes based on the similarity of the orthologs. First, we removed the short sequences (< 200 bp) from each sample, and reciprocal BLAST was used to remove the redundant orthologs (retained one-to-one orthologous expression levels). Then, the eight orthologous sequences were blasted using ClustalW 2.0, and the genes that are not located in the same section of a CDS was removed. Next, we deleted the redundant sequences at both ends, which were not colinear, using PERL. Finally, we linked the co-expressed genes into one long sequence. These eight long sequences represent the whole transcriptome of the parents and two hybrids that is expressed in the testis tissues. MEGA 5 was used to build the phylogenetic tree using Maximum likelihood tree (ML) methods.

### Dosage compensation in the two types of hybrids

To effectively analyze the dosage effects in the two types of hybrids, we analyzed the differential expression across the allodiploid and triploid hybrids compared with that in the middle parent in silico (MPVs). The MPVs were constructed from two parts, i.e., the maternal BSB value of gene expression (χ BSB) and the paternal YB values of gene expression (χ YB). The values were calculated as MPV1 = χ BSB + 1/2χ YB, MPV2 = 1/2χ BSB + 1/2 χYB and MPV3 = 1/2χ BSB + χ YB. An absolute value of the log 2 ratio ≤ 1.0 was used as the threshold to determine the significance of the gene expression differences. Expression values above the threshold were considered upregulated and those below the threshold were considered downregulated.

### Quantitative real-time PCR analysis

To determination the ELD and the HEB, several coexpressed genes were selected for validation using quantitative real-time PCR (qPCR) (primers are displayed in Additional file 1: Table S3). Total RNA was extracted from testicular tissues with the Trizol Reagent (Invitrogen) method, following the manufacturer’s protocol, and the first-strand cDNA was synthesized using a PrimeScript RT reagent Kit (Code: RR047A, TAKARA, China), with a PrimeScript RT Enzyme, at 37 °C for 15 min and 85 °C for 5 s. Actin gene (ACTB) was used as the internal control for the normalization of gene expression, and at least three independent biological samples and three technical replicates of each biological sample were analyzed by qPCR to ensure reproducibility and reliability. qPCR was performed using an ABI Prism 7500 Sequence Detection System (Applied Biosystems, USA). The amplification conditions were as follows: 50 °C for 5 min, 95 °C for 10 min, and 40 cycles at 95 °C for 15 s and 60 °C for 60 s. Relative quantification was performed, and a melting curve analysis was used to verify the generation of a single product at the end of the assay. The relative mRNA expression data were determined using the 2^-ΔΔCt^ method. Finally, the expression data were analyzed using Sigmaplot 12.5.

## Additional files

Additional file 1: **Table S1.** Summary of the annotated information of the testis transcriptome. **Table S2.** Bases counts distributed among the eight orthologs. **Table S3.** Special primers used for the verification of several genes (DOCX 18 kb)

Additional file 2: Data of the eight linked orthologs sequences. (XLSX 3757 kb)

Additional file 3: Expression level and annotated information of coexpressed unigenes between the parents and two types of hybrids. (XLSX 3190 kb)

Additional file 4: Annotations and GO functions of ELD genes in both types of hybrids. (XLSX 273 kb)

Additional file 5: Several growth-related genes showed BSB-ELD in the two types of hybrids. (DOCX 15 kb)

Additional file 6: Several chimeras verified in genome DNA level. (DOCX 235 kb)

Additional file 7: **Figure S1.** Effective primers for four homoeolog expression bias genes. (PNG 69 kb)

## Abbreviations

2nBY: Allodiploid hybrid
3nBY: Triploid hybrid
BSB: *Megalobrama amblycephala* Yih
DEGs: Differentially expression genes
ELD: Expression level dominance
HEB: Homoeolog expression bias
MPVs: Middle parent in silico
qPCR: Quantitative real-time PCR
SNPs: Single nucleotide polymorphisms
YB: *Xenocypri davidi* Bleeker
