# Supplementary material for: Asymmetric expression patterns reveal a strong maternal effect and dosage compensation in polyploid hybrid fish
Source: BMC Genomics. 2018 Jul 3;19:517. doi: 10.1186/s12864-018-4883-7 (PMC6030793; doi:10.1186/s12864-018-4883-7)
Supplement: Supplementary file 1 — Table S1. Summary of the annotated information of the testis transcriptome. Table S2. Bases counts distributed among the eight orthologs. Table S3. Special primers used for the verification of several genes (DOCX 18 kb) [file 12864_2018_4883_MOESM1_ESM.docx]

**Table S1 Summary of the annotated information of the testis transcriptome**

| Terms | Merge sample  Number (%) | BSB7  Number (%) | YB3  Number (%) | 2nBY3  Number (%) | 3nBY3  Number (%) |
| --- | --- | --- | --- | --- | --- |
| Annotated in NR | 86314(17.27%) | 47420(60.82) | 39887(50.18) | 58230(56.92) | 46295(57.00) |
| Annotated in NT | 344645(68.97%) | 65089(83.48) | 61971(77.97) | 81615(79.78) | 65474(39.75) |
| Annotated in KEGG | 31595(6.32%) | 34276(43.96) | 27305(34.35) | 41359(40.43) | 32284(39.75) |
| Annotated in SwissProt | 60657(12.14%) | 41527(53.26) | 33702(42.40) | 50451(49.32) | 39851(49.07) |
| Annotated in PFAM | 78232(15.65%) | - | - | - | - |
| Annotated in GO | 80234(16.05%) | 31586(40.51) | 25578(32.18) | 38322(37.46) | 30489(37.54) |
| Annotated in KOG | 21084(4.21%) | 13974(17.922) | 10339(13.00) | 15861(15.50) | 12488(15.38) |
| Annotated in at least one Database | 364543(72.96%) | 67038(85.98) | 64086(80.63) | 84558(82.66) | 67623(83.27) |
| Total unigenes | 499631 | 77,971 | 79,482 | 102,298 | 81,212 |

**Table S2 Bases counts distributed among the eight orthologs**

|  | Number | % | Number | % | Number | % | Number | % | A+T | % | G+C | (%) |
| --- | --- | --- | --- | --- | --- | --- | --- | --- | --- | --- | --- | --- |
| BSB | 699497 | 24.66 | 692841 | 24.42 | 721623 | 25.44 | 722683 | 25.48 | 1392338 | 49.08 | 1444306 | 50.92 |
| 3nBY1 | 699335 | 24.66 | 692841 | 24.43 | 721714 | 25.44 | 722558 | 25.47 | 1392176 | 49.09 | 1444272 | 50.92 |
| 3nBY2 | 700236 | 24.67 | 694072 | 24.45 | 721604 | 25.42 | 722631 | 25.46 | 1394308 | 49.12 | 1444235 | 50.88 |
| 3nBY3 | 700195 | 24.67 | 693659 | 24.44 | 721465 | 25.42 | 722415 | 25.46 | 1393854 | 49.11 | 1443880 | 50.89 |
| 2nBY1 | 700182 | 24.69 | 693401 | 24.45 | 720978 | 25.42 | 721665 | 25.44 | 1393583 | 49.14 | 1442643 | 50.86 |
| 2nBY2 | 701311 | 24.71 | 694272 | 24.46 | 720879 | 25.40 | 721640 | 25.43 | 1395583 | 49.17 | 1442519 | 50.83 |
| 2nBY3 | 701134 | 24.7 | 694531 | 24.47 | 720722 | 25.39 | 721744 | 25.43 | 1395665 | 49.18 | 1442466 | 50.82 |
| YB | 701104 | 24.73 | 694361 | 24.49 | 719796 | 25.38 | 720296 | 25.40 | 1395465 | 49.21 | 1440092 | 50.79 |

**Table S3 Special primers used for the verification of several genes**

| **Genes** | **Forward primer (5’-3’)** | **Reverse primer (5’-3’)** |
| --- | --- | --- |
| *ppia* | AACGGCAATCCACTTGGAAGAG | CCACCAGTTCCATCGTGTTTCG |
|  | GACGGCAAACCAGTTGGAAGAC | GCCAGTTCCATTGTGGTTCGTG |
| *lrp5* | TGATTTCTGTCATAAAGAGGAGCCC | TAATGGAGGCTTCCCACACGAGT |
|  | TCCTGTCACATGGTTTCTGTCGTAG | CAACGGAGGCTTCCCACATGAG |
| *syvn1* | ATTGGAAGCCAGGTTACAGTGCC | CGCCATCCTCATCTCCCTCCTT |
|  | TTTGGAAGCTAGGTTGCAGTGC | CTGCCATCCTCTTCTTCCTCCTT |
| *alg13* | CCGGGAACCATGTTGCTAGAGAC | GATAACTACTCTTTCAGGTCACGAAGG |
|  | TGGAACCATGGGGCTGGAGAC | TCTTTCAGGTCACGGAGTCGTC |
| *bmp2r* | GGGTTATCACCACGGATACACT | GGTCCATTCCACAGTTCACATCAG |
| *igf1* | ATAGTTTCTGCCCCCTGTGTTTC | CATTGTGGACGAATGCTGCTTTC |
| *mstn* | TTCGGCTGGGACTGGATTATTG | CAGCGGTCTACTACCATTGAGGG |
| *igf2* | CGAAAGTTTAGGAGGCAGGTGG | TTGACAAAGACAGTGGGCGAGT |
