# Supplementary material for: Asymmetric expression patterns reveal a strong maternal effect and dosage compensation in polyploid hybrid fish
Source: BMC Genomics. 2018 Jul 3;19:517. doi: 10.1186/s12864-018-4883-7 (PMC6030793; doi:10.1186/s12864-018-4883-7)
Supplement: Supplementary file 5 — Several growth-related genes showed BSB-ELD in the two types of hybrids. (DOCX 15 kb) [file 12864_2018_4883_MOESM5_ESM.docx]

| Gene_ID | Genes  Name | Discription | KO | Length  (bp) | 2nBY_T1  (FPKM) | 2nBY_T2  (FPKM) | 2nBY_T3  (FPKM) | YB_T1  (FPKM) | YB_T2  (FPKM) | YB_T3  (FPKM) | 3nBY_T1  (FPKM) | 3nBY_T2  (FPKM) | 3nBY_T3  (FPKM) | BSB1_T1  (FPKM) | BSB1_T2  (FPKM) | BSB1_T3  (FPKM) |
| --- | --- | --- | --- | --- | --- | --- | --- | --- | --- | --- | --- | --- | --- | --- | --- | --- |
| TRINITY_DN155220_c6_g1 | *GPC4* | Glypican 4 | K08110 | 2310 | 7.34 | 7.49 | 6.73 | 1.1 | 1.8 | 2.74 | 15.6 | 15.32 | 7.05 | 18.54 | 11.08 | 13.15 |
| TRINITY_DN145506_c1_g1 | *stat3* | Signal transducer and activator of transcription | K04692 | 3952 | 15.54 | 15.19 | 22.42 | 3.99 | 4.44 | 10.19 | 26.97 | 31.68 | 17.28 | 30.09 | 25.26 | 22.71 |
| TRINITY_DN117251_c0_g2 | *GATA3* | Transcription factor GATA-3 | K17895 | 986 | 8.49 | 5.22 | 2.98 | 44.56 | 23.18 | 18.09 | 4.79 | 5.78 | 6.8 | 3.56 | 3.65 | 3.7 |
| TRINITY_DN155371_c11_g1 | *MGAT5* | Mannoside Acetylglucosaminyltransferase 5 | K00744 | 11252 | 21.61 | 22.09 | 28.69 | 14.9 | 11.09 | 8.1 | 37.75 | 32.98 | 25.42 | 28.38 | 25.33 | 28.33 |
| TRINITY_DN146251_c1_g1 | *RYK* | Receptor-like tyrosine kinase | K05128 | 3615 | 106.12 | 145.93 | 88.82 | 27.19 | 26.97 | 30.51 | 73.17 | 81.72 | 63.21 | 134.19 | 120.74 | 126.22 |
| TRINITY_DN139264_c3_g1 | *VDAC2* | Vdac2 protein | K15040 | 1623 | 104.92 | 104.73 | 176.64 | 23.93 | 24.56 | 73.47 | 131.58 | 119.08 | 85.76 | 145.19 | 112.68 | 96.58 |
| TRINITY_DN131545_c2_g1 | *FZD2* | Frizzled class receptor 2 | K02235 | 1594 | 16.01 | 19.43 | 16.72 | 7.66 | 8.69 | 7.27 | 19.03 | 17.04 | 10.59 | 20.67 | 21.63 | 16.46 |
| TRINITY_DN151854_c4_g1 | *PIM1* | Serine/threonine-protein kinase pim-2 | K04702 | 2215 | 59.34 | 71.19 | 26.38 | 37.85 | 48.2 | 43.84 | 41.12 | 49.35 | 46.23 | 56.22 | 58.69 | 29.4 |
| TRINITY_DN139199_c3_g3 | *MSH2* | DNA mismatch repair protein | K08735 | 3391 | 138.88 | 132.87 | 52.66 | 37.42 | 37.21 | 40.82 | 212.95 | 187.52 | 83.41 | 125.24 | 124.43 | 72.81 |
| TRINITY_DN148219_c0_g2 | *MELK* | Maternal embryonic leucine zipper kinase | K08799 | 2930 | 33.62 | 26.36 | 18.09 | 32.46 | 28.92 | 10.09 | 25.56 | 16.64 | 12.21 | 12.27 | 14.21 | 8.83 |
| TRINITY_DN127947_c1_g1 | *BCL2L10* | BCL2-like 10 (Apoptosis facilitator) | K18451 | 1412 | 78.12 | 68.81 | 134.53 | 135.48 | 123.13 | 75.76 | 38.87 | 44.51 | 45.97 | 30.3 | 34.51 | 29.81 |
| TRINITY_DN137291_c1_g1 | *THOC6* | THO complex subunit 6 homolog | K13175 | 2252 | 39.59 | 43.89 | 49.05 | 14 | 8.79 | 22.24 | 42.72 | 33.41 | 44.92 | 51.15 | 44.54 | 43.98 |
| TRINITY_DN154189_c5_g1 | *RAD50* | RAD50 Double Strand Break Repair Protein | K10866 | 1170 | 261.57 | 279.79 | 188.44 | 392.86 | 454.17 | 205.56 | 166.37 | 164.23 | 143.1 | 118.7 | 117.06 | 110.47 |
| TRINITY_DN120411_c1_g2 | *CDH1* | Cadherin 1, type 1, E-cadherin | K03364 | 1303 | 10.37 | 6.69 | 7.61 | 13.69 | 13.28 | 11.82 | 10.11 | 9.32 | 14.58 | 11.02 | 13 | 13.62 |
| TRINITY_DN132424_c3_g2 | *STYX* | Serine/threonine/tyrosine-interacting protein | K18042 | 906 | 43.47 | 45.99 | 30.28 | 15.95 | 14.59 | 35.93 | 54.57 | 51.77 | 56.05 | 81.07 | 65.18 | 60.32 |
