# Supplementary material for: Asymmetric expression patterns reveal a strong maternal effect and dosage compensation in polyploid hybrid fish
Source: BMC Genomics. 2018 Jul 3;19:517. doi: 10.1186/s12864-018-4883-7 (PMC6030793; doi:10.1186/s12864-018-4883-7)

1. *Myo18b (partial genome DNA sequence)*


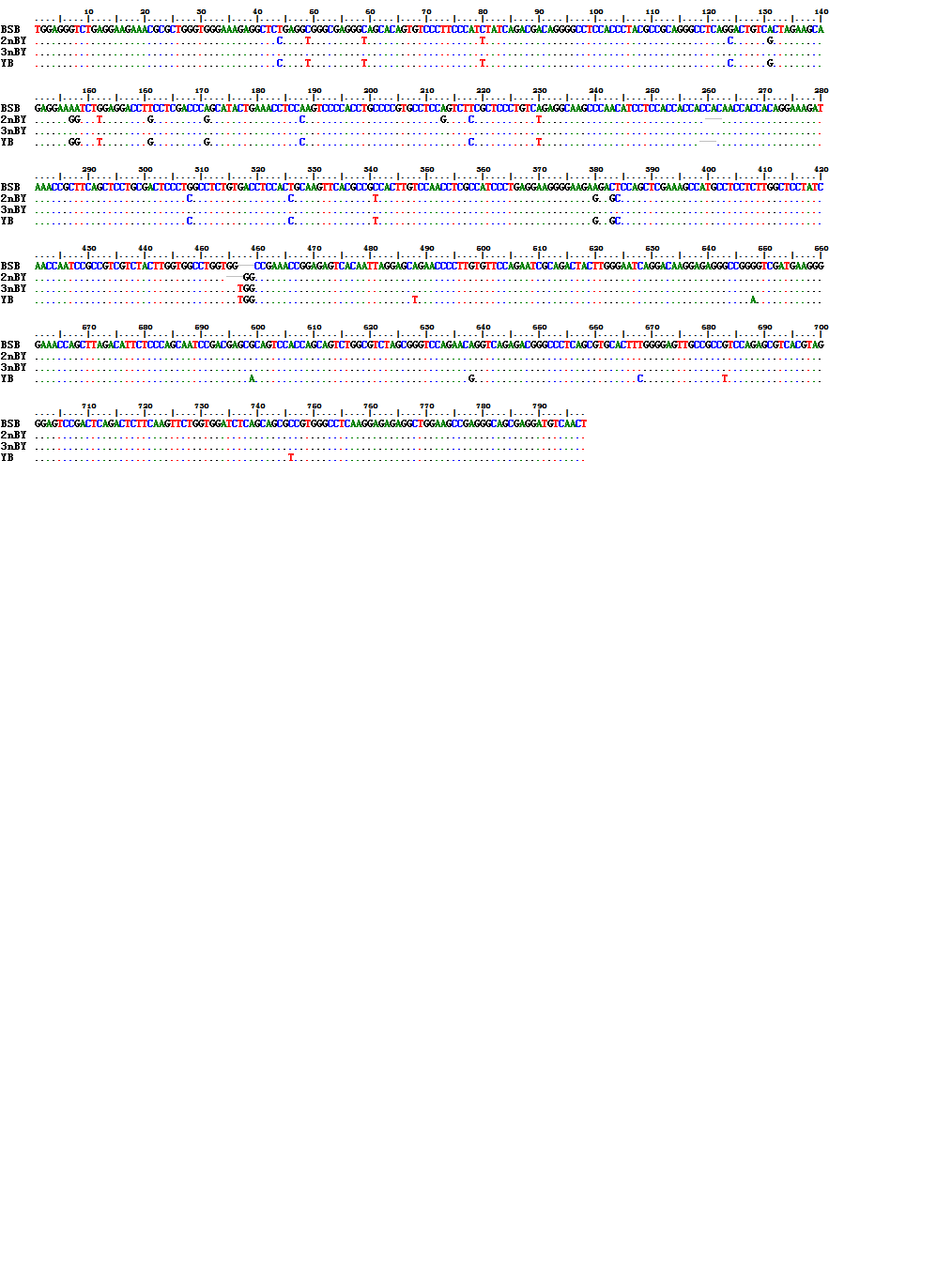


1. *tiss22 (partial genome DNA sequence)*


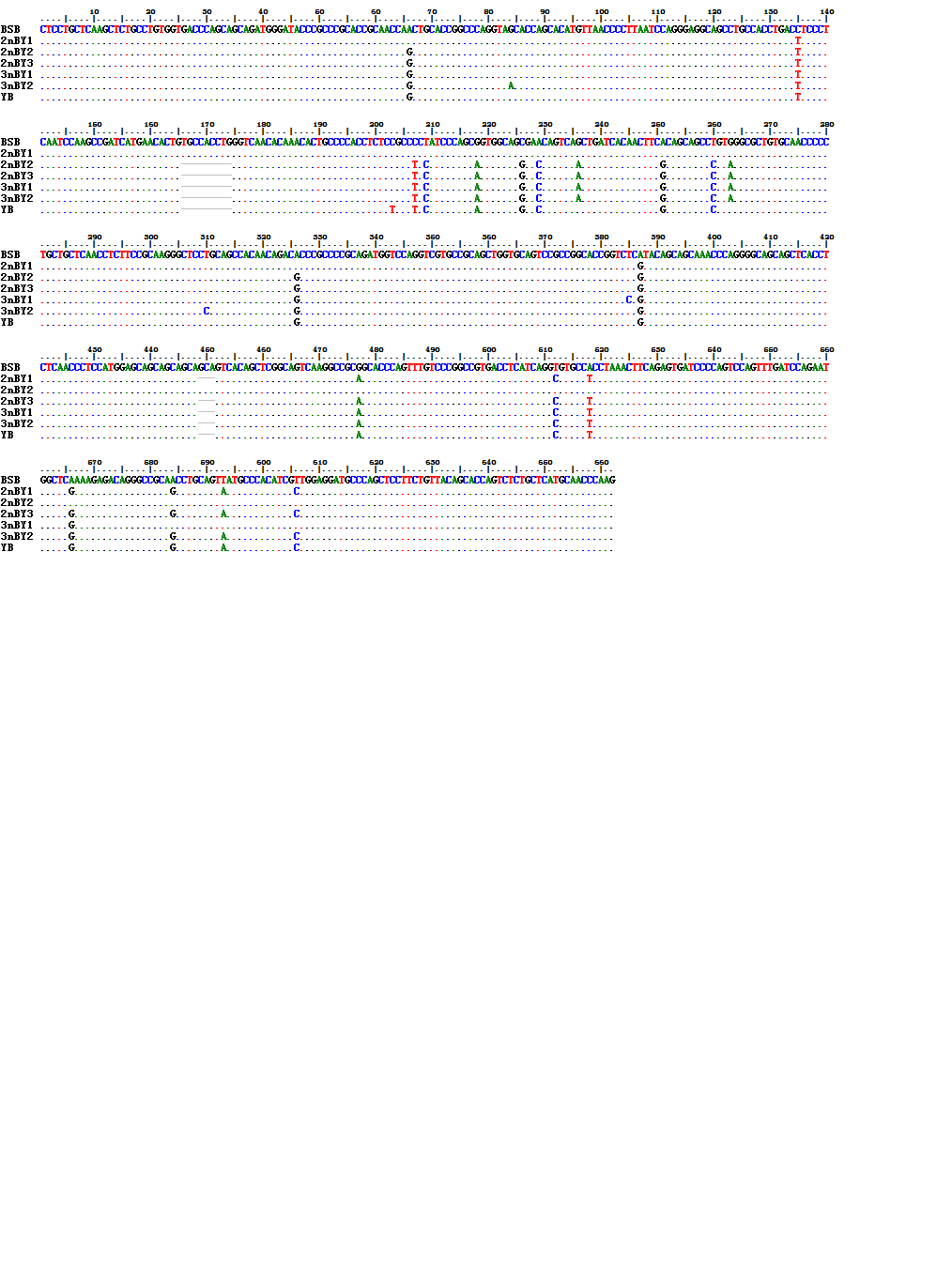


1. *Fitm (partial genome DNA sequence)*


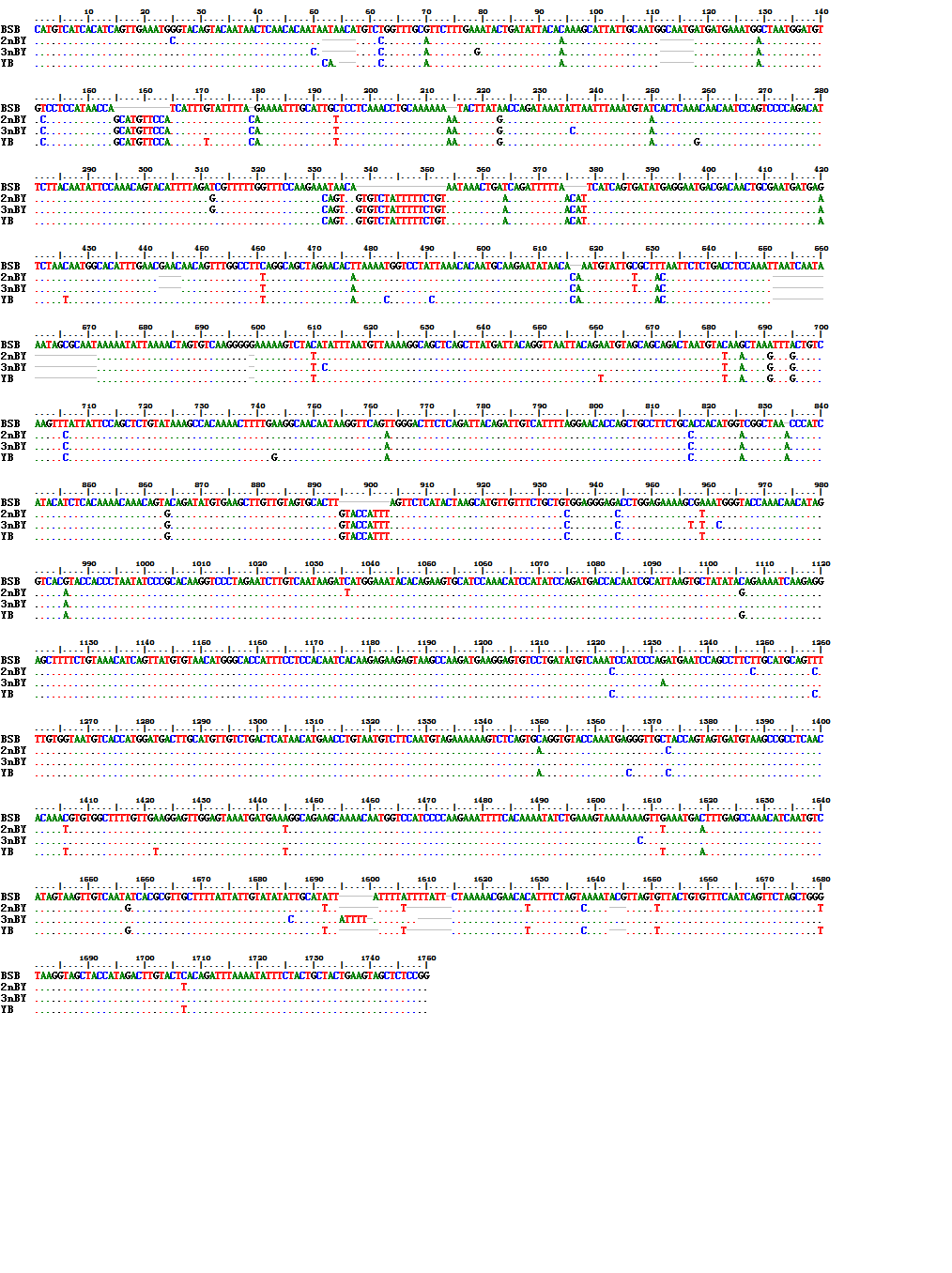


1. *Ptpn (partial genome DNA sequence)*


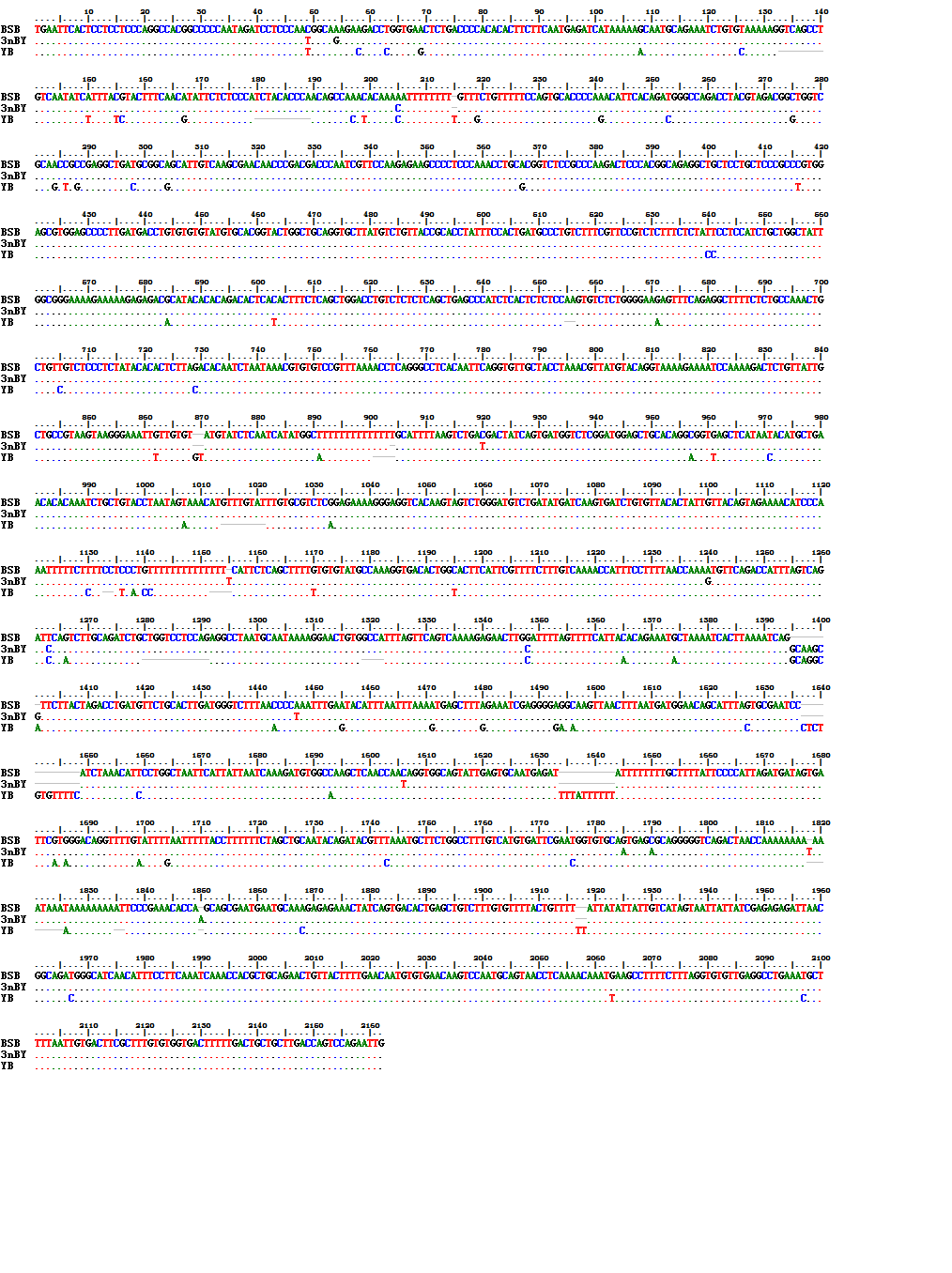

Supplement: Supplementary file 6 — Several chimeras verified in genome DNA level. (DOCX 235 kb) [file 12864_2018_4883_MOESM6_ESM.docx]
