# Supplementary figures and images for: Asymmetric expression patterns reveal a strong maternal effect and dosage compensation in polyploid hybrid fish
Source: BMC Genomics. 2018 Jul 3;19:517. doi: 10.1186/s12864-018-4883-7 (PMC6030793; doi:10.1186/s12864-018-4883-7)

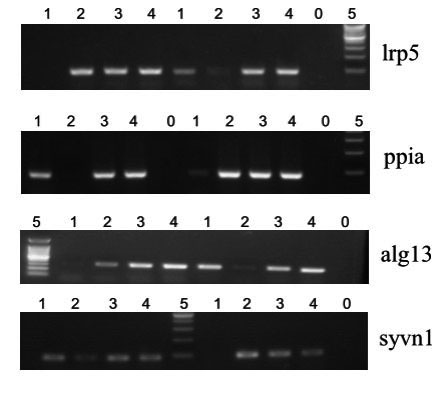

Supplement: Supplementary file 7 — Figure S1. Effective primers for four homoeolog expression bias genes. (PNG 69 kb) [file 12864_2018_4883_MOESM7_ESM.png]
